# Burden of AML, 1990-2019: Estimates From the Global Burden of Disease Study

**DOI:** 10.1200/GO.23.00229

**Published:** 2023-11-22

**Authors:** Chinmay T. Jani, Alaaeldin Ahmed, Harpreet Singh, Christian Mouchati, Omar Al Omari, Padmanabh S. Bhatt, Rajesh Sharma, Minaam Farooq, Weitao Liu, Joseph Shalhoub, Dominic Marshall, Justin D. Salciccioli, Jeremy L. Warner, Prudence Lam

**Affiliations:** ^1^ Sylvester Comprehensive Cancer Center at University of Miami, Miami, FL; ^2^ Department of Medicine, Mount Auburn Hospital, Harvard Medical School, Cambridge, MA; ^3^ Department of Medicine, Harvard Medical School, Boston, MA; ^4^ M.D.R Collaborative Group, London, United Kingdom; ^5^ Department of Pulmonary and Critical Care, Medical College of Wisconsin, Milwaukee, WI; ^6^ Department of Neurology, Cleveland Clinic, Cleveland, OH; ^7^ Department of Pulmonary and Critical Care, Temple University, Philadelphia, PA; ^8^ Humanities and Social Science, National Institute of Technology Kurukshetra, Haryana, India; ^9^ King Edward Medical University, Lahore, Pakistan; ^10^ Academic Section of Vascular Surgery, Department of Surgery and Cancer, Imperial College London, London, United Kingdom; ^11^ Critical Care Research Group, Nuffield Department of Clinical Neurosciences, University of Oxford, Oxford, United Kingdom; ^12^ Department of Pulmonary and Critical Care, Brigham and Women's Hospital, Harvard Medical School, Boston, MA; ^13^ Center for Clinical Cancer Informatics and Data Science, Legorreta Cancer Center, Brown University, Providence, RI; ^14^ Lifespan Cancer Institute, Rhode Island Hospital, Providence, RI; ^15^ Division of Hematology and Oncology, Mount Auburn Hospital, Harvard Medical School, Cambridge, MA

## Abstract

**PURPOSE:**

AML accounts for 80% of acute leukemia in adults. While progress has been made in treating younger patients in the past 2 decades, there has been limited improvement for older patients until recently. This study examines the global and European Union (EU) 15+ trends in AML between 1990 and 2019.

**METHODS:**

We extracted age-standardized incidence rates (ASIRs), age-standardized death rates (ASMRs), and disability-adjusted life years, stratified by sex from the Global Burden of Disease Study database, and mortality-to-incidence ratio (MIR) were computed. Trends were compared using Joinpoint regression.

**RESULTS:**

The findings show a global increase in AML incidence for both sexes from 1990 to 2019. In the EU15+ countries, most countries exhibited an increase in ASIR for both sexes. Joinpoint revealed that globally for male patients, ASIR steadily increased until 2010, remained stable until 2015 followed by a decline till 2019. Similar trends were observed in female patients. For ASMR, although there was an increase globally and in most EU15+ countries, there was a statistically significant decrease in mortality rates globally and in the majority of EU15+ countries in recent years. MIR improved in both sexes globally. On age stratification, AML burden was highest among older groups (55 years and older), while the lowest rates were observed in younger than 20 years.

**CONCLUSION:**

The findings from our study indicate a global rise in AML incidence and mortality in both sexes and decrease in MIR from 1990 to 2019 suggesting a better survival. However, on Joinpoint analysis, there is no change in MIR in women in the past decade and past 4 years in men indicating plateau in survival trends despite recent advances.

## INTRODUCTION

AML is the most common acute leukemia in adults and is responsible for approximately 80% of all patients of acute leukemias.^1^ In adults, AML is a disease of the aged, with a median age at diagnosis of 68 years.^2^ Globally, the annual number of newly diagnosed patients of all leukemias has increased by 46% in the past 3 decades. The major responsible factors are aging, as well as an increase in secondary leukemias due to the widespread use of cytotoxic chemotherapy.^3^ Incidence for all leukemias has consistently decreased by 0.93% per year in the past 3 decades. However, incidence for AML has increased by 15%, and the proportion of AML accounting for total patients with leukemia has increased by 27%.^1^ With the highest percentage of deaths (60%), it is one of the deadliest leukemias. Outcomes are worst in the subset of patients who are aged 65 years, in which only 30% survive at 1-year intervals.^4-6^

**CONTEXT**

**Key Objective**
This study investigates global and European Union (EU) 15+ trends in AML between 1990 and 2019.
**Knowledge Generated**
This study provides insights into AML epidemiology, including age-standardized incidence rates (ASIRs), age-standardized death rates (ASMRs), disability-adjusted life years, and mortality-to-incidence ratio (MIR). Globally, AML incidence increased for both sexes, with most EU15+ countries showing rising ASIR. However, a shift occurred, with ASIR rising until 2010, stabilizing until 2015, and declining from 2015 to 2019 globally. Despite a global ASMR increase, many EU15+ countries saw significant mortality rate reductions. MIR improved globally, indicating better survival. AML burden was highest among older age groups (55 years and older) and lowest among those younger than 20 years.
**Relevance**
This study provides a comprehensive analysis of AML epidemiology, highlighting a global rise in incidence and mortality but also improved survival trends, with some recent plateauing. Continued research and advancements in treatment are warranted.

The management approach of AML remained relatively stagnant for decades. However, in the past 5 years, considerable therapeutic progress has been made in understanding molecular and genetic pathogenesis and testing, along with the development of novel targeted therapies.^7^ These advances in diagnostics, therapeutics, risk stratification, and supportive care of AML are expected to improve AML-associated disability and mortality.^8,9^ However, these advances are yet to be implemented in low- or middle-income countries.

The main objective of this study was to compare the trends in age-standardized incidence rates (ASIRs), age-standardized death rates (ASMRs), mortality-to-incidence ratios (MIRs), and disability-adjusted life years (DALYs) because of AML globally and European Union (EU) 15+ countries from 1990 to 2019. EU15+ comprises Austria, Belgium, Canada, Denmark, Finland, France, Germany, Greece, Ireland, Italy, Luxembourg, Netherlands, Norway, Portugal, Spain, and Sweden. With Australia, the United Kingdom, and the United States, this group represents a readily comparable group of 19 countries because of similar health infrastructure^10,11^ and completeness of critical registration reporting.^12,13^ Regions were divided into Africa, Americas, South-East Asia, European, Eastern Mediterranean, and Western Pacific, as described by the WHO.^14^ To our knowledge, no such recent analysis comparing trends in this cohort of countries has been performed.

## METHODS

### Characteristics of the Data Source

This observational analysis of AML among EU15+ countries was performed using data from the Global Burden of Disease (GBD) database. We have used this method to describe trends in intracerebral hemorrhage (ICH),^15^ thyroid cancer,^16^ and kidney cancer.^17^ For AML data, the GBD maps data related to the International Classification of Diseases (ICD) codes (code C92 and its descendants from ICD, 10th Revision [ICD-10] and code 205 and its descendants from ICD, Ninth Revision [ICD-9]). These data are then combined by Bayesian metaregression with the DisMod-MR 2.19 tool that analyzes, adjusts for bias, and produces disease estimates with uncertainty intervals.

The data from vital registration sources, verbal autopsy reports, and surveillance data are used as input to arrive at mortality estimates using the cause-of-death ensemble model and are entered into the GBD cause-of-death database. The quality of mortality data from each country are rated by the GBD in a 5-star system by location year to assist in the reader's comprehension of the reliability of the cause of death data. The EU15+ countries have been previously analyzed this way, with 10 of 19 scoring five stars (85%-100% completeness of mortality data), and the remaining nine countries scoring four stars (65%-84% completeness of mortality data).^17^

### Handling of the GBD Data

We used the GBD study results tool to extract ASIR, ASMRs, and DALYs data for AML between 1990 and 2019.^18^ GBD calculates a standard population from the United Nations Population Division's World Population Prospects.^19^ Subsequently, absolute and relative changes were calculated for each sex in each country. The MIR was calculated by dividing ASMR by ASIR. DALYs incorporate morbidity and mortality figures to calculate the number of years lived with and lost from a disability. The WHO uses them to indicate the overall disease burden on a health system.^20^ Mean trends of global and different WHO region were also reported for comparison.

### Statistical Analysis

Joinpoint Command Line version 4.5.0.1 was used to apply a Joinpoint regression analysis (provided by the US National Cancer Institute Surveillance Research Program).^21^ The software observes trends in the data over the study period and connects these trends with the simplest model possible on a logarithmic scale. It will identify specific inflection points in the overall trends and provide a robust estimate of changing trends. It computes estimated annual percentage change (EAPC) for each trend and tests for significance using a Monte Carlo permutation method.

### Subgroup Analysis

Age-stratified analysis was performed by dividing the entire population on the basis of available age groups in the GBD database: younger than 20 years, 20-54, and 55 and older.

## RESULTS

### Trends in AML Incidence

**FIG 1:**
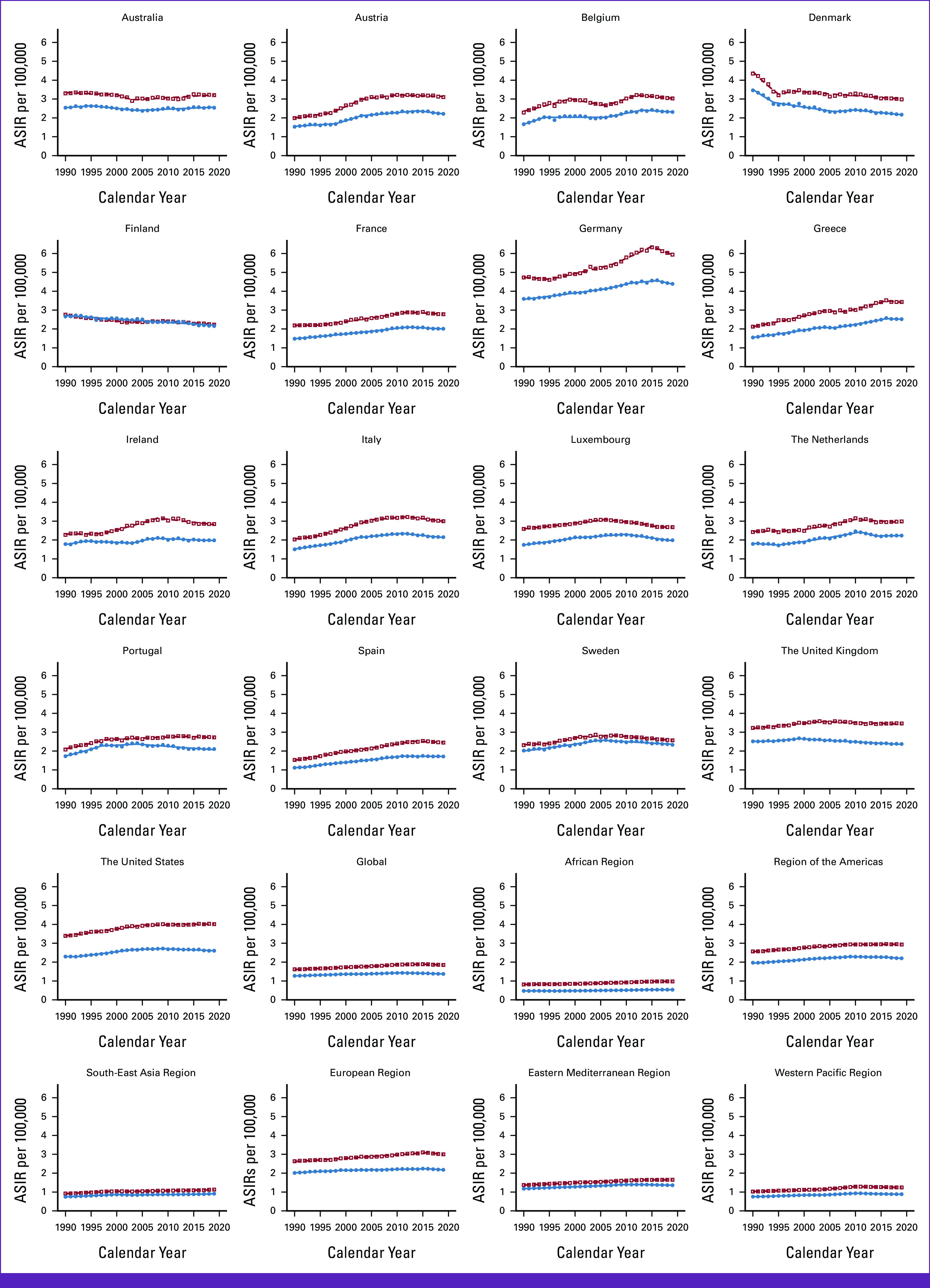
Trends in ASIRs per 100,000 for AML Globally, in WHO regions and European Union 15+ countries between 1990 and 2019. Open squares indicate male patients, and filled circles indicate female patients. ASIR, age-standardized incidence rate.

Globally, ASIR has increased for male patients (+34.60%) and female patients (+7.9%; Tables 1 and 2 and Fig 1). In EU15+ countries, 15/19 (79%) countries showed an increase in ASIR for male patients, and 14/19 (74%) countries showed an increase for female patients. In 2019, Germany had the highest ASIR for male patients (5.94/100,000) and female patients (4.39/100,000), and the lowest ASIR was observed in Norway for male patients (2.00/100,000) and female patients (1.37/100,000).

**TABLE 1:**
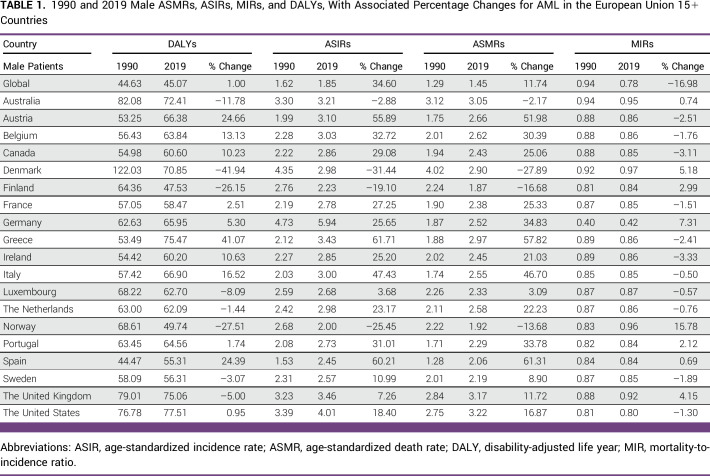
1990 and 2019 Male ASMRs, ASIRs, MIRs, and DALYs, With Associated Percentage Changes for AML in the European Union 15+ Countries

**TABLE 2:**
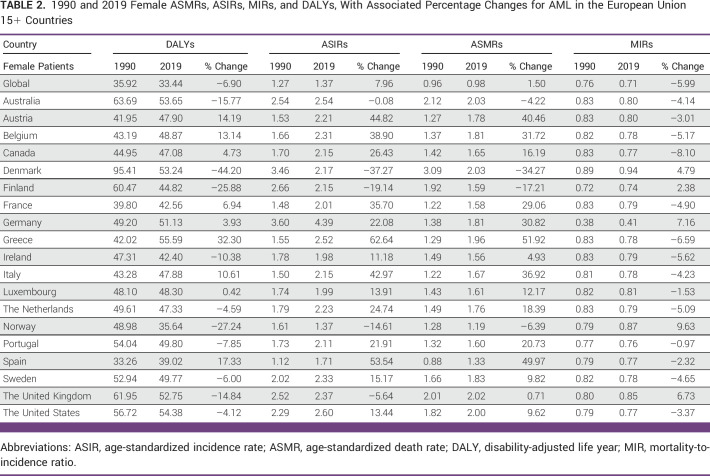
1990 and 2019 Female ASMRs, ASIRs, MIRs, and DALYs, With Associated Percentage Changes for AML in the European Union 15+ Countries

For male patients, globally, ASIR steadily increased until 2010 (EAPC, 0.7%; 1990-2007 and 1.1%; 2007-2010; Data Supplement, eTable 1A). It was followed by a flat trend from 2010 to 2015 (0.2% [0%-0.5%]) and then a decline till 2019 (–0.6%). Among EU15+ countries, latest EAPC were either decreasing 12/19 (63%) or flat in 6/19 (32%) except Australia (+0.5% [2003-2019]). The highest decrease was seen in Germany from 2015 to 2019 (–1.8%) in male patients.

For female patients, globally, ASIR increased till 2011, followed by a steady decline till 2019 (–0.5%; Data Supplement, eTable 1B). Among EU15+ countries, latest EAPC were decreasing in 13/19 (68%) or flat in 5/19 (26%) except Australia (+0.5% [2004-2019]). However, the greatest decrease was seen in Luxemburg (–1.8% [2010-2019]).

### Trends in AML Mortality

**FIG 2:**
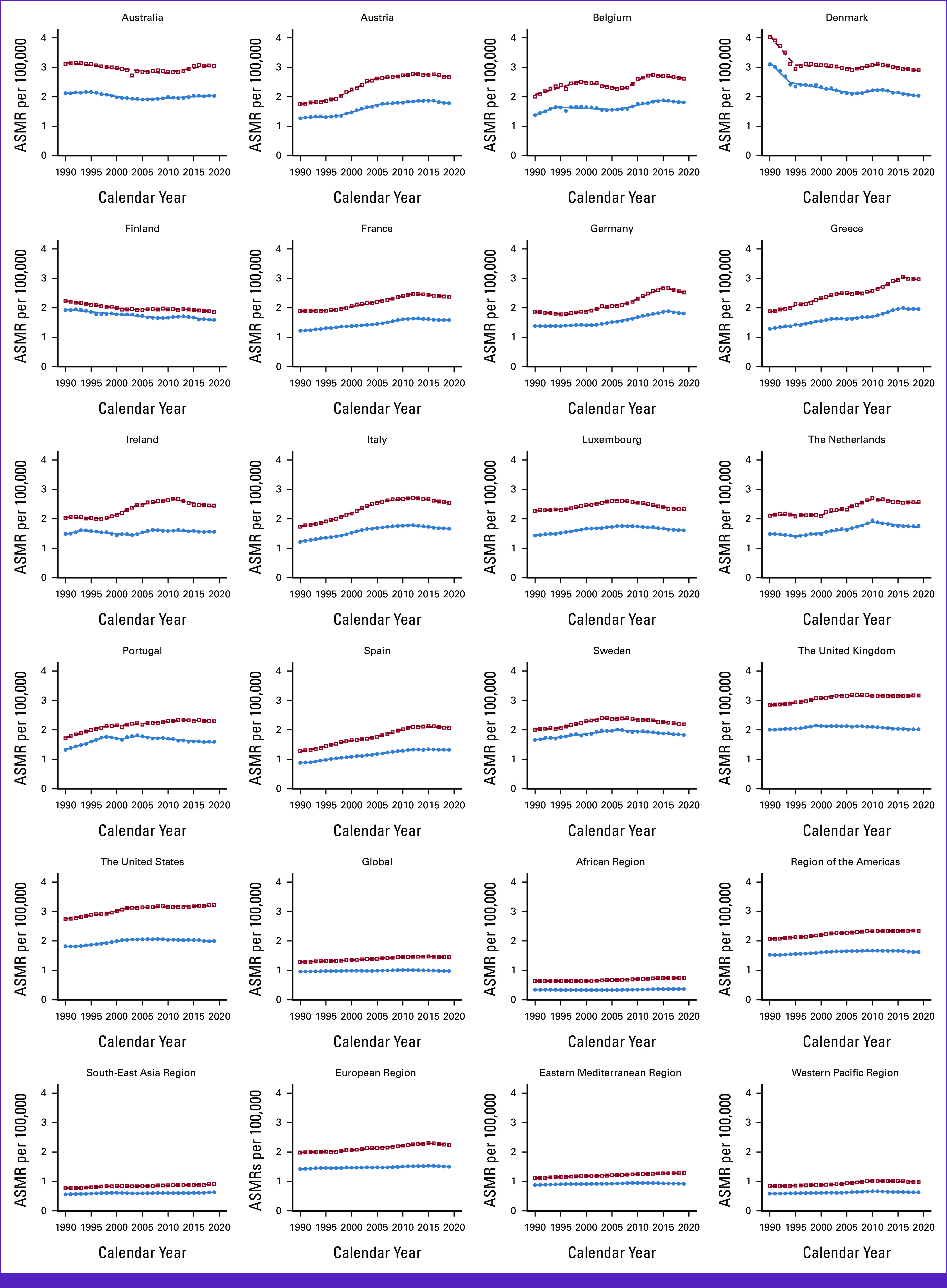
Trends in ASMRs per 100,000 for AML Globally, in WHO regions and European Union 15+ countries between 1990 and 2019. Open squares indicate male patients, and filled circles indicate female patients. ASMR, age-standardized mortality rate.

Globally, ASMR has increased for male patients (+11.74%) and female patients (+1.50%; Tables 1 and 2 and Fig 2). In EU15+ countries, 15/19 countries showed an increase in ASMR for male patients, and 15/19 countries showed an increase for female patients. In 2019, the United States had the highest ASMR for male patients (+3.22/100,000), and Denmark had the highest ASMR for female patients (+2.03/100,000). The lowest ASMR was observed in Finland for male patients (+1.87/100,000) and Norway for female patients (+1.19/100,000).

For male patients, globally, EAPC for ASMR steadily increased till 2011 (0.4% [1990-1997] and 0.7% [1997-2011]; Data Supplement, eTable 2A). Trend remained flat from 2011 to 2015 (0.1%) and then declined till 2019 (–0.5%). Among EU15+ countries, the latest EAPC decreased in 12/19 (63%) countries and steady in 5/19 (26%). Discordant latest trends were observed in the United States (+0.2% [2002-2019]) and Australia (+1 [2009-2019]). The greatest latest decrease was observed in Germany (–1.7% [–2.7% to 0.7%]) from 2015 to 2019.

For female patients, globally, EAPC for ASMR steadily increased till 2011. ASMR declined from 2011 to 2019 (–0.5%; Data Supplement, eTable 2B). Among EU15+ countries, latest EAPC were decreasing in 14/19 (74%) and flat in 3/19 (16%). Worsening latest mortality trends were observed in Norway (+0.5 [2004-2019]) and Australia (+0.5 [2004-2019]). The greatest decrease was observed in Germany (–1.7% [2010-2019]).

### Trends in AML MIR

Globally, MIR has decreased for male patients (–16.98%) and female patients (–5.99%; Tables 1 and 2). In EU15+ countries, 11/19 countries showed a decrease in MIR for male patients, and 14/19 countries showed a decrease for female patients. In 2019, the MIR was the highest in Denmark for male patients (+0.97/100,000) and female patients (+0.94/100,000). Germany had the lowest MIR for male patients (+0.42/100,000) and female patients (+0.41/100,000).

For male patients, globally, EAPC for MIR had four trends with a steady decline from 1990 to 1999 and 2005 to 2015 (–0.2% and –0.1%), respectively (Data Supplement, eTable 3A). However, the recent trend from 2015 to 2019 remained flat (+0.1%). Among EU15+ countries, latest EAPCs were flat in 14/19 (74%). The highest increase was seen in Norway (+0.4% [2006-2019]).

For female patients, globally, MIR steadily decreased till 2010 and has not changed since (0% [2010-2019]; Data Supplement, eTable 3B). Among EU15+ countries, the latest EAPC were flat in 11/19 (58%) and increasing in 8/19 (42%). Highest increases were seen in Luxemburg (+0.8% [2011-2019]) and in Norway (+0.8% [1998-2019]).

### Trends in AML DALYs

**FIG 3:**
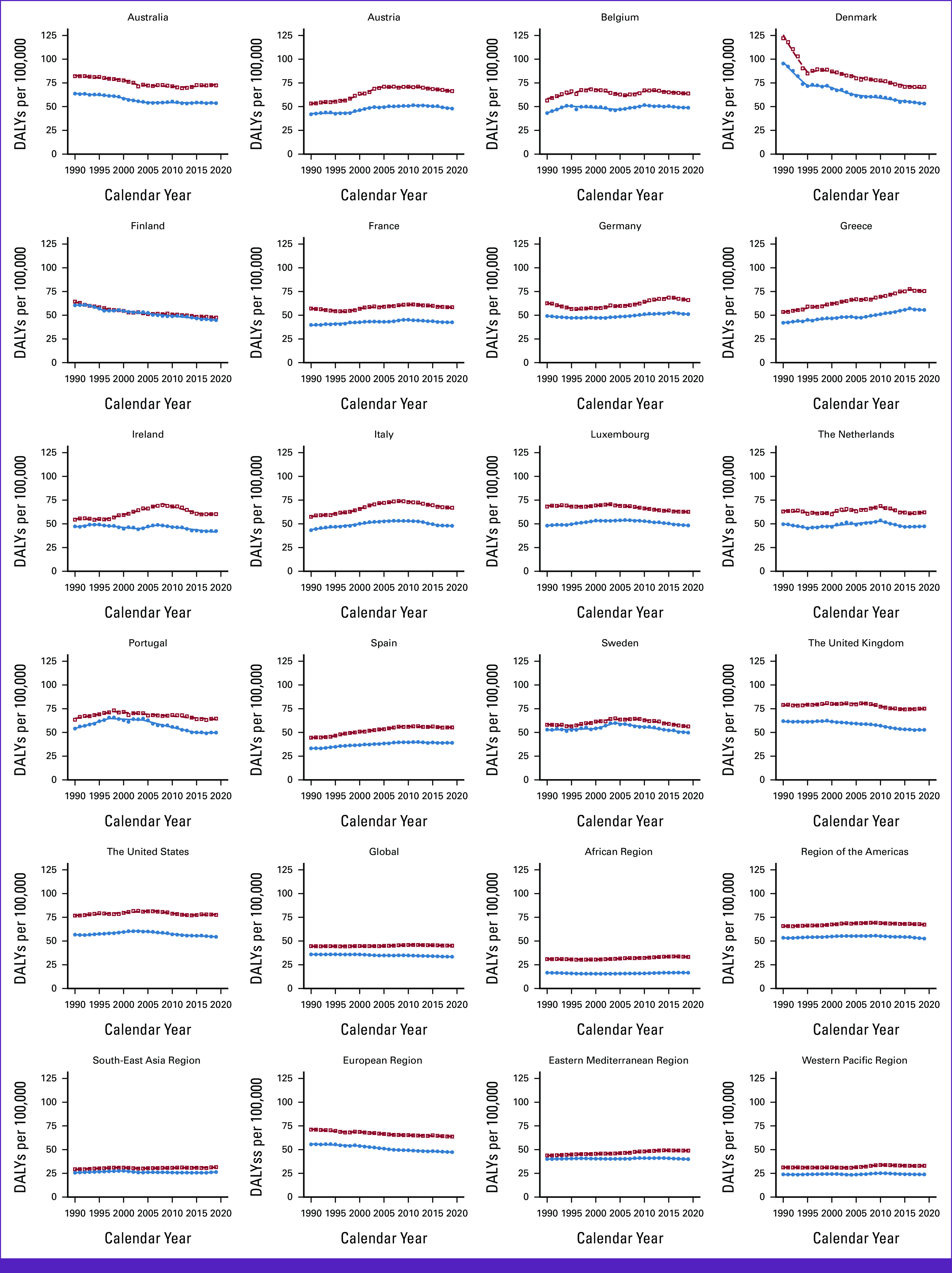
Trends in DALYs per 100,000 for AML Globally, in WHO regions and European Union 15+ countries between 1990 and 2019. Open squares indicate male patients, and filled circles indicate female patients. DALY, disability-adjusted life year.

DALYs have increased for male patients (+1.00%) but decreased for female patients globally (–6.90%; Tables 1 and 2; Fig 3). In EU15+ countries, 11/19 countries showed an increase in DALYs for male patients, and 9/19 countries showed an increase for female patients. The United States had the highest DALYs for male patients (+77.51/100,000), and Greece had the highest DALYs for female patients (+55.59/100,000). In 2019, the lowest DALYs were observed in Finland for male patients (+47.53/100,000) and Norway for female patients (+35.64/100,000).

For male patients, globally, EAPC for DALYs was observed to have a flat trend from 1990 to 2004, a steady increase till 2011, followed by a decline since (–0.2 [2011-2019]; Data Supplement, eTable 4A). Among EU15+ countries, the latest EAPC decreased in 12/19 (63%) and was statistically unchanged in 6/16 (31%). In recent years, an increase in DALYs in men was observed in Australia only (+0.6%). The highest decreases were seen in Sweden and Norway with the same rate (–1.3% [2009-2019]) for male patients.

For female patients, globally, EAPC for DALYs was observed to have four Joinpoint trends during the study period with a decrease in the past decade (–0.4% [2009-2019]; Data Supplement, eTable 4B). Among EU15+ countries, the latest EAPC decreased in 12/19 (63%) and remained steady in 7/19 (37%) for female patients. The highest decrease was seen in Ireland for female patients (–1.4% [2007-2019]).

### Subgroup Analysis With Age Stratification

#### Age Group Younger than 20

The lowest ASIR, ASMR, and DALYs were observed in this age group (Data Supplement, eTables 4 and 7). Globally, there was a decrease in all parameters for both sexes. ASMR, ASIR, and DALYs decreased for all EU15+ countries except Greece. Greece saw an increase in DALYs for female patients (+2.18%), an increase in ASIR for male patients (+2.37%), and female patients (+15.95%), as well as ASMR for female patients (+2.30%). MIR decreased for 15/19 countries for male patients and female patients.

#### Age Group 20-54 Years

The second highest ASIR, ASMR, and DALYs were observed in 20-54 years (Data Supplement, eTables 5 and 8). Globally, except for a decrease in DALYs (–1.47%) and MIR (–7.00) for female patients, there was an increase in DALYs and MIR for male patients and ASIR and ASMR for both sexes. For EU15+ countries, ASIR was increasing in 11/19 countries for male patients and 9/19 for female patients, and ASMR was increasing for 8/19 countries for male patients and 9/19 for female patients. However, MIR was found to increase in 5/19 countries for male patients and 8/19 for female patients. A similar trend was found for DALYs, increasing in 8/19 countries for male patients and 7/19 for female patients.

#### Age Group 55 Years and Older

The highest burden of ASIR, ASMR, and DALYs was observed in this age group (Data Supplement, eTables 6 and 9). Globally, there was an increase in ASIR, ASMR, and DALYs. However, MIR decreased in male patients (–8.97%) and female patients (–7.05%). Among EU15+ countries, ASIR and ASMR increased for all countries except Norway and Denmark for male patients and Finland and Denmark for female patients. DALYs increased in 14/19 countries for male patients and 16/19 countries for female patients. Although there was a rising burden in this age group, MIR decreased in all countries for female patients and 15/19 for male patients.

## DISCUSSION

The findings from our study indicate a global rise in AML incidence in both sexes from 1990 to 2019. Similarly, in EU15+ countries, most of the countries had an overall increase in the incidence. At the same time, ASMR in most EU15+ countries have decreased in the past decade except in the United States and Australia, which have discordant trends with EAPC of 0.2% and 1%, respectively. In 2019, we noted that the United States had the highest mortality rate as measured by ASMR for male patients while Denmark had the highest for female patients (2.03/100,000), followed by Australia (2.031/100,000), the United Kingdom (2.02/100,000), and the United States (2.00/100,000). All these countries had higher ASIR as well. Among all countries, the Unites States had the second-highest male ASIR (4.01/100,000), followed by the United Kingdom (3.46/100,000) and Australia (3.21/100,000). These higher incidences can be one of the contributory factors to higher ASMR in these countries. This finding is interesting as early disease detection is critical for improved survival.

In contrast to overall trends, there has been no change in MIR in women in the past decade and past 4 years in men. Similarly, MIR trends have remained unchanged in most EU15+ countries in recent years. Many patients with AML are idiopathic, with genetic predisposition, ionization radiation, cytotoxic chemotherapy/drugs, herbicides, and pesticides all having been linked to AML.^22,23^ A better understanding of the molecular pathogenesis of AML led to a more precise selection of management strategies. Considerable therapeutic progress has been made in novel target therapies that interact with the AML responsible gene changes. Despite this promising progress, unchanged MIR in the past decade in EU15+ countries indicates that the mortality outcomes in AML remain stagnant despite recent advances. This is likely due—at least in part—to the relatively recent rollout of some of these therapies and the inevitable lag in clinical adoption after regulatory approvals are granted. For example, although venetoclax was approved by the US Food and Drug Administration (FDA) and European Medicines Agency (EMA) in 2016, it did not acquire an AML label by FDA until 2018 (accelerated approval) and did not receive full FDA approval for AML until 2020.^24^ It is also observed that EMA tends to approve cancer therapy later than FDA, with a median delay of 241 days.^25^

The higher incidence, mortality, DALYs, and MIR are observed in men in all the EU15+ countries indicating higher disease burden and lower survival rates in men. Disproportionate disease burden in men has been reported for most cancer types, including hematological malignancies.^26,27^ Previous studies have shown that higher AML disease burden is not consistent over all age groups, and the sex gap narrows in the older population.^28^ However, our study revealed consistent sex disparities across different age groups: younger than 20 years, 20-54 years, and 55 years and older, emphasizing the persistent impact on men. The increased disease burden in men can likely be attributed to factors such as higher rates of smoking, alcohol consumption, and greater exposure to environmental and occupational hazards.^29-32^

Like other cancers, smoking has been identified as contributors to both the incidence and mortality of AML.^22^ In most EU15+ countries, the prevalence of smoking has either stabilized or decreased. A similar trend is observed in the United States, where smoking prevalence has declined by 6% over the past 15 years.^33^ The stabilization of ASIR in recent years across most EU15+ countries, despite an increase in the proportion of older adults and a rising prevalence of obesity worldwide (especially in EU15+ countries), might be explained by changes in smoking prevalence.^34,35^ Trends of ASIR in the United States from the SEER database and Canadian studies mirror our results.^36,37^ However, another US study reported a 1.5-2 times increase in the ASIR from AML from 2011 to 2018.^38^

Apart from smoking, the aging population and obesity are significant risk factors contributing to the development of AML, with a marked increase in risk associated with higher BMI levels, especially class II and III obesity.^39^ Genetic alterations (TET2, JAK2, and ASXL1) have been linked to age-related clonal hematopoiesis, a condition commonly found in healthy individuals that becomes more prevalent with advancing age and is considered a precursor to AML.^1,40^ Studies have also indicated a connection between AML development in individuals aged 60 and older and a history of smoking. Researchers estimated that 40% of patients with AML with maturation had developed smoking-induced leukemia.^41^ In our age-stratified analysis, we found that ASIR, ASMR, and DALYs were highest in the age group of 55 and older. Conversely, incidence and mortality decreased in the younger population (younger than 20). However, there was a global and widespread increase in older age groups (55 years and older), indicating an overall rise in disease burden among the elderly population. When examining these results in EU15+ countries, the United Kingdom displayed the highest ASMR in male patients aged 55 and older (16.92/100,000), followed by Australia (15.95/100,000) and the United States (15.66/100,000). However, when assessing their mortality rates in younger age groups, all three countries reported ASMR values that were either lower than or equal to the median ASMR values in their respective age groups. These findings indicate that these countries carry a heavier burden of AML within their older population.

The GBD study collaborators are transparent regarding the limitations of using the GBD database, and we have previously discussed them.^15,16^ Notable limitations include alterations in data coding systems and country-specific practice, including a transition from ICD-9 to ICD-10 over the study period. By mapping mortalities to causes of death lists, the GBD authors adjust to the different coding systems. Second, variability exists within and across countries in the accuracy of death certification with errors in death certification ranging from 39% to 61% worldwide.^11,42,43^ However, the top-performing continents in relation to civil registration and vital statistics were Europe, the Americas, and Australasia,^10^ which augments the reliability of the data presented from EU15+ countries in this study. The GBD uses under-registration corrections and garbage-code distribution algorithms to adjust for under-registration.^44,45^ Garbage codes relate to deaths resulting from poorly defined diagnoses or those that cannot be the single underlying cause of death. For the subgroup analysis, the age division is not uniform because of data availability. In addition, our analysis mainly represents high-income countries, and therefore, the external validity is low when applying to other countries belonging to lower income and developing world. In addition, as with all observational analyses, there are likely contributory confounders that are not fully accounted for. Finally, we stress that this is an observational analysis from which causal inferences should not be concluded.

In conclusion, this study provides population-based trends in AML epidemiology inclusive of all ages and age-stratified subgroup analysis. The findings from our study indicate a global rise in AML incidence and mortality in both sexes and decrease in MIR from 1990 to 2019 suggesting a better survival. However, on Joinpoint analysis, there is no change in MIR in the past decade in women and in past 4 years in men, indicating a plateau in survival trends despite recent advances.

## References

[1] Dong Y Shi O Zeng Q, et al: Leukemia incidence trends at the global, regional, and national level between 1990 and 2017. Exp Hematol Oncol 9:14, 2020 32577323 10.1186/s40164-020-00170-6 PMC7304189

[2] Cancer Stat Facts: Leukemia—Acute Myeloid Leukemia (AML). National Cancer Institute Surveillance, Epidemiology, and End Results Program. https://seer.cancer.gov/statfacts/html/amyl.html

[3] Martinez-Cuadron D Megias-Vericat JE Serrano J, et al: Treatment patterns and outcomes of 2310 patients with secondary acute myeloid leukemia: A PETHEMA registry study. Blood Adv 6:1278-1295, 2022 34794172 10.1182/bloodadvances.2021005335 PMC8864639

[4] Ferlay J Soerjomataram I Dikshit R, et al: Cancer incidence and mortality worldwide: Sources, methods and major patterns in GLOBOCAN 2012. Int J Cancer 136:E359-E386, 2015 25220842 10.1002/ijc.29210

[5] Siegel RL Miller KD Jemal A: Cancer statistics, 2016. CA Cancer J Clin 66:7-30, 2016 26742998 10.3322/caac.21332

[6] Nachtkamp K Stark R Strupp C, et al: Causes of death in 2877 patients with myelodysplastic syndromes. Ann Hematol 95:937-944, 2016 27025507 10.1007/s00277-016-2649-3

[7] Stanchina M Soong D Zheng-Lin B, et al: Advances in acute myeloid leukemia: Recently approved therapies and drugs in development. Cancers (Basel) 12:3225, 2020 33139625 10.3390/cancers12113225 PMC7692236

[8] Bower H Andersson TM Bjorkholm M, et al: Continued improvement in survival of acute myeloid leukemia patients: An application of the loss in expectation of life. Blood Cancer J 6:e390, 2016 26849011 10.1038/bcj.2016.3 PMC4771966

[9] DiNardo CD Jonas BA Pullarkat V, et al: Azacitidine and venetoclax in previously untreated acute myeloid leukemia. N Engl J Med 383:617-629, 2020 32786187 10.1056/NEJMoa2012971

[10] Mikkelsen L Phillips DE AbouZahr C, et al: A global assessment of civil registration and vital statistics systems: Monitoring data quality and progress. Lancet 386:1395-1406, 2015 25971218 10.1016/S0140-6736(15)60171-4

[11] Lu TH Shau WY Shih TP, et al: Factors associated with errors in death certificate completion. A national study in Taiwan. J Clin Epidemiol 54:232-238, 2001 11223320 10.1016/s0895-4356(00)00299-7

[12] Murray CJ: Quantifying the burden of disease: The technical basis for disability-adjusted life years. Bull World Health Organ 72:429-445, 1994. 8062401 PMC2486718

[13] Murray CJ Acharya AK: Understanding DALYs (disability-adjusted life years). J Health Econ 16:703-730, 1997 10176780 10.1016/s0167-6296(97)00004-0

[14] Joinpoint Trend Analysis Software. National Cancer Institute—Division of Cancer Control & Population Sciences. https://surveillance.cancer.gov/joinpoint/

[15] DeLago AJ Jr Singh H Jani C, et al: An observational epidemiological study to analyze intracerebral hemorrhage across the United States: Incidence and mortality trends from 1990 to 2017. J Stroke Cerebrovasc Dis 31:106216, 2022 35091266 10.1016/j.jstrokecerebrovasdis.2021.106216

[16] Schuster-Bruce J Jani C Goodall R, et al: A comparison of the burden of thyroid cancer among the European Union 15+ countries, 1990-2019: Estimates from the global burden of disease study. JAMA Otolaryngol Head Neck Surg 148:350-359, 2022 35266977 10.1001/jamaoto.2021.4549 PMC8914910

[17] Jani C Abdallah N Mouchati C, et al: Trends of kidney cancer burden from 1990 to 2019 in European Union 15 + countries and World Health Organization regions. Sci Rep 12:22368, 2022 36572700 10.1038/s41598-022-25485-8 PMC9792551

[18] GBD Results Tool. http://ghdx.healthdata.org/gbd-results-tool

[19] World Population Prospects: The 2012 Revision Highlights and Advance Tables. UN Department of Economic and Social Affairs. https://population.un.org/wpp/publications/Files/WPP2012_HIGHLIGHTS.pdf

[20] World Health Organization: Metrics: Disability-Adjusted Life Year (DALY). https://www.who.int/healthinfo/global_burden_disease/metrics_daly/en/

[21] Kim HJ Fay MP Feuer EJ, et al: Permutation tests for joinpoint regression with applications to cancer rates. Stat Med 19:335-351, 2000 10649300 10.1002/(sici)1097-0258(20000215)19:3<335::aid-sim336>3.0.co;2-z

[22] Choi E Lee S Nhung BC, et al: Cancer mortality-to-incidence ratio as an indicator of cancer management outcomes in Organization for Economic Cooperation and Development countries. Epidemiol Health 39:e2017006, 2017 28171715 10.4178/epih.e2017006 PMC5434228

[23] Dohner H Weisdorf DJ Bloomfield CD: Acute myeloid leukemia. N Engl J Med 373:1136-1152, 2015 26376137 10.1056/NEJMra1406184

[24] FDA: FDA Grants Regular Approval to Venetoclax in Combination for Untreated Acute Myeloid Leukemia. https://www.fda.gov/drugs/resources-information-approved-drugs/fda-grants-regular-approval-venetoclax-combination-untreated-acute-myeloid-leukemia

[25] Lythgoe MP Desai A Gyawali B, et al: Cancer therapy approval timings, review speed, and publication of pivotal registration trials in the US and Europe, 2010-2019. JAMA Netw Open 5:e2216183, 2022 35687337 10.1001/jamanetworkopen.2022.16183 PMC9187952

[26] Parkin DM Bray F Ferlay J, et al: Global cancer statistics, 2002. CA Cancer J Clin 55:74-108, 2005 15761078 10.3322/canjclin.55.2.74

[27] Cook MB Dawsey SM Freedman ND, et al: Sex disparities in cancer incidence by period and age. Cancer Epidemiol Biomarkers Prev 18:1174-1182, 2009 19293308 10.1158/1055-9965.EPI-08-1118 PMC2793271

[28] Sant M Allemani C Tereanu C, et al: Incidence of hematologic malignancies in Europe by morphologic subtype: Results of the HAEMACARE project. Blood 116:3724-3734, 2010 20664057 10.1182/blood-2010-05-282632

[29] Lee DJ Voti L MacKinnon J, et al: Gender- and race-specific comparison of tobacco-associated cancer incidence trends in Florida with SEER regional cancer incidence data. Cancer Causes Control 19:711-723, 2008 18322816 10.1007/s10552-008-9134-9

[30] Hauptmann M Lubin JH Stewart PA, et al: Mortality from lymphohematopoietic malignancies among workers in formaldehyde industries. J Natl Cancer Inst 95:1615-1623, 2003 14600094 10.1093/jnci/djg083

[31] Merhi M Raynal H Cahuzac E, et al: Occupational exposure to pesticides and risk of hematopoietic cancers: meta-analysis of case-control studies. Cancer Causes Control 18:1209-1226, 2007 17874193 10.1007/s10552-007-9061-1

[32] Bassil KL Vakil C Sanborn M, et al: Cancer health effects of pesticides: Systematic review. Can Fam Physician 53:1704-1711, 2007. 17934034 PMC2231435

[33] WHO Regional Office for Europe: Tobacco: Data and Statistics. http://www.euro.who.int/en/health-topics/disease-prevention/tobacco/data-and-statistics

[34] Cesare M Bentham J Stevens G, et al: Trends in adult body-mass index in 200 countries from 1975 to 2014: A pooled analysis of 1698 population-based measurement studies with 19·2 million participants. Lancet 387:1377-1396, 2016 27115820 10.1016/S0140-6736(16)30054-X PMC7615134

[35] WHO Regional Office for Europe: WHO European Regional Obesity Report 2022. World Health Organization, 2022. https://www.who.int/europe/publications/i/item/9789289057738

[36] Howlader N Noone AM Krapcho M, et al: SEER Cancer Statistics Review, 1975–2016. Bethesda, MD, National Cancer Institute, 2019

[37] Shysh AC Nguyen LT Guo M, et al: The incidence of acute myeloid leukemia in Calgary, Alberta, Canada: A retrospective cohort study. BMC Public Health 18:94, 2017 28774275 10.1186/s12889-017-4644-6 PMC5543578

[38] Hao T Li-Talley M Buck A, et al: An emerging trend of rapid increase of leukemia but not all cancers in the aging population in the United States. Sci Rep 9:12070, 2019 31427635 10.1038/s41598-019-48445-1 PMC6700310

[39] Poynter JN Richardson M Blair CK, et al: Obesity over the life course and risk of acute myeloid leukemia and myelodysplastic syndromes. Cancer Epidemiol 40:134-140, 2016 26720913 10.1016/j.canep.2015.12.005 PMC4738058

[40] Bowman RL Busque L Levine RL: Clonal hematopoiesis and evolution to hematopoietic malignancies. Cell Stem Cell 22:157-170, 2018 29395053 10.1016/j.stem.2018.01.011 PMC5804896

[41] Fircanis S Merriam P Khan N, et al: The relation between cigarette smoking and risk of acute myeloid leukemia: An updated meta-analysis of epidemiological studies. Am J Hematol 89:E125-E132, 2014 24753145 10.1002/ajh.23744

[42] Burger EH van der Merwe L Volmink J: Errors in the completion of the death notification form. S Afr Med J 97:1077-1081, 2007 18250917

[43] Katsakiori PF Panagiotopoulou EC Sakellaropoulos GC, et al: Errors in death certificates in a rural area of Greece. Rural Remote Health 7:822, 2007 18067402

[44] GBD 2017 Causes of Death Collaborators: Global, regional, and national age-sex-specific mortality for 282 causes of death in 195 countries and territories, 1980-2017: A systematic analysis for the Global Burden of Disease Study 2017. Lancet 392:1736-1788, 2018 30496103 10.1016/S0140-6736(18)32203-7 PMC6227606

[45] GBD 2017 Disease and Injury Incidence and Prevalence Collaborators: Global, regional, and national incidence, prevalence, and years lived with disability for 354 diseases and injuries for 195 countries and territories, 1990-2017: A systematic analysis for the Global Burden of Disease Study 2017. Lancet 392:1789-1858, 2018 30496104 10.1016/S0140-6736(18)32279-7 PMC6227754

